# One Health communication channels: a qualitative case study of swine influenza in Canada in 2020

**DOI:** 10.1186/s12889-024-18460-7

**Published:** 2024-04-05

**Authors:** José Denis-Robichaud, Suzanne Hindmarch, Nancy N. Nswal, Jean Claude Mutabazi, Mireille D’Astous, Marcellin Gangbè, Andrea Osborn, Christina Zarowsky, Erin E. Rees, Hélène Carabin

**Affiliations:** 1 Université de Montréal, QC Montréal, Canada; 2 University of New Brunswick, NB Fredericton, Canada; 3 Public Health Agency of Canada, QC Saint-Hyacinthe, Canada; 4 Canadian Food Inspection Agency, BC Parksville, Canada; 5 Centre de Recherche en Santé Publique, QC Montréal, Canada; 6 Groupe de recherche en épidémiologie des zoonoses et santé publique, Saint-Hyacinthe, QC Canada

**Keywords:** Public health, Information dissemination, Agriculture, Disease outbreak, Zoonoses, Health services, Veterinarians

## Abstract

**Background:**

With increased attention to the importance of integrating the One Health approach into zoonotic disease surveillance and response, a greater understanding of the mechanisms to support effective communication and information sharing across animal and human health sectors is needed. The objectives of this qualitative case study were to describe the communication channels used between human and animal health stakeholders and to identify the elements that have enabled the integration of the One Health approach.

**Methods:**

We combined documentary research with interviews with fifteen stakeholders to map the communication channels used in human and swine influenza surveillance in Alberta, Canada, as well as in the response to a human case of H1N2v in 2020. A thematic analysis of the interviews was also used to identify the barriers and facilitators to communication among stakeholders from the animal and human health sectors.

**Results:**

When a human case of swine influenza emerged, the response led by the provincial Chief Medical Officer of Health involved players at various levels of government and in the human and animal health sectors. The collaboration of public and animal health laboratories and of the swine sector, in addition to the information available through the surveillance systems in place, was swift and effective. Elements identified as enabling smooth communication between the human and animal health systems included preexisting relationships between the various stakeholders, a relationship of trust between them (e.g., the swine sector and their perception of government structures), the presence of stakeholders acting as permanent liaisons between the ministries of health and agriculture, and stakeholders' understanding of the importance of the One Health approach.

**Conclusions:**

Information flows through formal and informal channels and both structural and relational features that can support rapid and effective communication in infectious disease surveillance and outbreak response.

## Background

Influenza virus surveillance and response to spillover between species are situations that can benefit from a One Health (OH) approach, as they occur at the intersection of animal, human, and ecosystem health sectors [1]. An improved understanding of intersectoral communication across OH domains is important because many emerging diseases are of animal origin [2], and many global forces (increased mobility of people, animals, animal products and goods, climate change, agribusiness expansion, deforestation, etc.) are increasingly altering environments to put animals and humans in close contact, facilitating disease spillover in both directions. Identifying formal and informal structures, processes or practices that support OH communication could improve the integration of the OH approach in different systems.

Human infections with swine influenza virus subtypes have been reported in North America [3, 4], and these are reportable under the International Health Regulations (IHR), although data suggest that transmission of influenza from humans to pigs is more frequent [5]. Here, we report a human case of influenza A H1N2v occurring in Alberta in October 2020. It resulted in rapid collaboration and investigation by human and animal health sectors, but there is limited information about how and why effective communication and coordination occurred between and within these sectors during this event. Bridging this gap requires gathering information from multiple points of view, to which qualitative methods are well suited [6]. Describing the context and narrative of a specific case study enables identification of patterns that can then be validated in other contexts. Our study objectives were to describe the OH communication channels and flow of information among stakeholders involved in human and swine influenza surveillance and response activities in Alberta (Canada) and to identify elements encouraging and inhibiting OH communication, specifically related to information sharing between livestock^1^ and public health professionals. Our research question was therefore to determine what factors impede or support information sharing between sectors during the occurrence of a human case of zoonotic influenza.

## Methods

To describe the mechanisms and performance of communication channels, we used interpretive process tracing [6–8]. We started from the detection of the emergence of a human case of influenza A H1N2v in Alberta in October 2020. We then sought to understand the communication channels related to the surveillance of influenza in pigs and humans generally and how these and other channels operated in this specific case. Our research team included animal and public health researchers and government employees, but none were directly involved in case management or regional surveillance systems related to this event. We relied on the experience and knowledge from our collaborator from the Animal Health Science Directorate of the Canadian Food Inspection Agency (CFIA) to identify some key stakeholders.

Documentary research and interviews with stakeholders influenced each other in an iterative process. The Canadian Animal Health Surveillance System (CAHSS) was a starting point for documentary research, as it already mapped the surveillance system within multiple animal production industries [9]. Additionally, we used a report created after the 2009 H1N1 pandemic [10] and a recent study about laboratory and syndromic surveillance in the swine sector [11] to create a preliminary outline of Canadian influenza communication channels.

We initially identified ten stakeholders occupying strategic positions in the case study communications channels, representing federal and provincial governments, animal and public health, and Canadian swine health surveillance systems. Additional stakeholders were identified through snowballing and findings from concurrent documentary research [12, 13]. Interviewees were invited to participate in a one-hour individual semistructured interview to identify and explore structural links and information channels.

We developed semistructured interview questions during the initial phases of the documentary research and created a general interview guide to identify the case study communication channels and the barriers and facilitators for communication between animal and human health stakeholders (Table 1). The guide was tested with a team member involved in animal health surveillance who did not participate in developing the questions. The data from this pilot were kept for the analyses. The research team met throughout the project to discuss and assess the guide and minimize biases [14]. For example, interviewers adapted the guide to make it relevant to each interviewed stakeholder by choosing questions that aligned with their work and position. Some questions were also rephrased or complemented to fill gaps identified during previous interviews. Changes and additions were reviewed by members of the research team, all of whom assessed the questions from their own disciplinary vantage point to ensure these alterations were consistent with the global objectives of the study and did not reflect the implicit assumptions or biases of any one discipline or sector. While the main interviewer was an animal health specialist, she was joined by at least one other member of the team from another discipline during all interviews. Having one interviewer with deep subject matter expertise ensured continuity and rigour across interviews; having a second research team member from a different disciplinary vantage point served as a check to reduce confirmation bias.

**Table 1:** General interview guide for stakeholders involved in a human case of influenza A H1N2v

**Topic**	**Questions and probes**
Introduction	- “To begin, can you tell us about your position and role? - “Thank you again for meeting us today. Our overall objective is to strengthen the use of information for decision making about health risks across human and animal health surveillance systems, in a One Health perspective. We want to understand how information is currently shared or not shared, and to document barriers, opportunities and structural links between and within animal and human health surveillance networks and systems in Canada. As a [role/position], your role is central for these networks, and we would like to understand your perspective and experience of how the information flows. - “To do so, we will briefly talk about the general structure and information flow in case of [animal/zoonotic] emergency health event, and then ask you to describe how it played out in the case of the case of influenza A H1N2v that happened in 2020.
Global structure and information flow	- “Can you first tell us how information about a possible emergency [animal/zoonotic] health event flows to you and from you? - What are your main sources of information? - Do you use GPHIN? CEZD? Social media? - “Once the information comes to you, what happens? How is the information shared? - “What determines whether the information is shared more broadly to [animal/public health] surveillance systems? - “What do you think could facilitate your work regarding sharing information in case of a possible [animal/zoonotic] health event?
Case study	
Transition	- “An example is likely going to help me better understand the specifics of the flow of information. If I am correct, you played a role when the influenza A H1N2v case was identified in a human patient who did not have contact with pigs.
Information flow	- “Can you tell us how you heard about this case first? - Was through an official channel? Can you explain how this works? - “What happened with this information? - How did you assess whether this information was a concern, something that needs to be shared with others so that it can be acted on? - What were the triggers for information sharing, and to whom? - What information was shared with other surveillance systems, agencies (e.g., public health, environmental health), experts (e.g., labs and universities)? - When? How? - How did you get additional information if needed? - If so, was it from different sources of information? Which ones? - Were you involved in discussion or work group(s) to address the issue? - What happened/what were the results of these?
Barriers and facilitators	- “Working in a bureaucratic system can have its challenges, were there elements that prevented you from obtaining or sharing key information? - “Were there elements that, on the other hand, helped the information to flow? - “What are the most important lessons that have been learned that you think would be important to use for future events? - “I bet a human case of swine influenza is not the only time you receive information about the flu. What is the flow of information for suspected or confirmed [swine] influenza cases in [pig farms/the population]? - How do you get this information? - Are you responsible for sharing this information? To whom? What are the triggers? - When is this information shared to [animal/public] health?
Wrap-up	- “Considering we want to document barriers, opportunities and structural links between, and within animal and human health surveillance networks and systems in Canada, is there anything I have not asked about, that you think is important for me to know? - “Are there people you think would be useful to interview about these objectives?

*GPHIN* Global Public Health Intelligence Network, *CEZD* Community for Emerging and Zoonotic Diseases

Interviews were conducted in English or French between September and December 2021, and (with permission) audio recorded on Zoom (Zoom Video Communications, Inc.) or Teams (Microsoft corp.). Interviews were transcribed and cleaned and then coded and analyzed in NVivo (Luminvero©). To protect anonymity, all interview quotes in this report are presented in English. Participants did not receive compensation. We contacted 23 stakeholders from the human (*n*=13) and animal health (*n*=10) sectors, of whom eight (human: *n* = 6, and animal: *n* = 2) declined or did not reply to our invitations (nonparticipation proportion = 35%). Fifteen participants from the human (*n* = 7) and animal (*n* = 8) health sectors were interviewed in November and December 2021. Employees of the federal (Public Health Agency of Canada and CFIA) and provincial (Alberta Health Services and Alberta Ministry of Agriculture) governments, stakeholders from the swine health surveillance system and from academia participated in the interviews (Table 2).

**Table 2:** Interviewee sectoral and jurisdictional location

**Sector**	**Provincial**	**Federal**	**Other**	**Total**
Human health	3	4	0	7
Animal health	2	3	3	8
**Total**	5	7	3	15

### Analyses

Through an interpretive process tracing approach, we explored how actors described their practices, how they perceived their actions, and how information flows [8, 15]. We used interview transcripts combined with documentary research to create a map of the communication channels among stakeholders involved in human and swine influenza surveillance in Alberta and in the specific H1N2v zoonotic human influenza case. We then synthesized this information graphically using an online collaborative platform (Miro; RealtimeBoard, Inc.). We identified two distinct categories of communication channels: formal and informal. Formal channels were those that entailed an institutionalized, official structure, often including established written protocols, guidance documents, or terms of reference specifying how actors holding specific positions of authority were to communicate with each other. Informal channels were ad hoc, created by the involved stakeholders to suit a particular situation, and were often dependent on personal relationships between individuals, rather than institutionalized relationships between offices or job functions. We used an iterative thematic analysis [16, 17] to identify barriers and facilitators to information sharing in this case. Themes and subthemes summarizing participants’ perspectives were discussed among members of our research team, and representative quotes were selected. We used this information about facilitators and barriers to identify elements that, more broadly, may support or impede information sharing between animal health and human health stakeholders.

## Results

While our study focused on a human case of swine influenza, it quickly became clear that the surveillance systems in place prior to the event were important. Surveillance systems have multiple goals. For influenza in Canada, surveillance aims to detect and monitor the viruses, and to inform vaccines and policies [18, 19].

### Routine communication structures

We describe below the usual communication channels in the swine sector, the human health sector, and across these two sectors.

#### Routine communication channels for the surveillance of influenza virus infection in the swine sector in Alberta

**Fig. 1:**
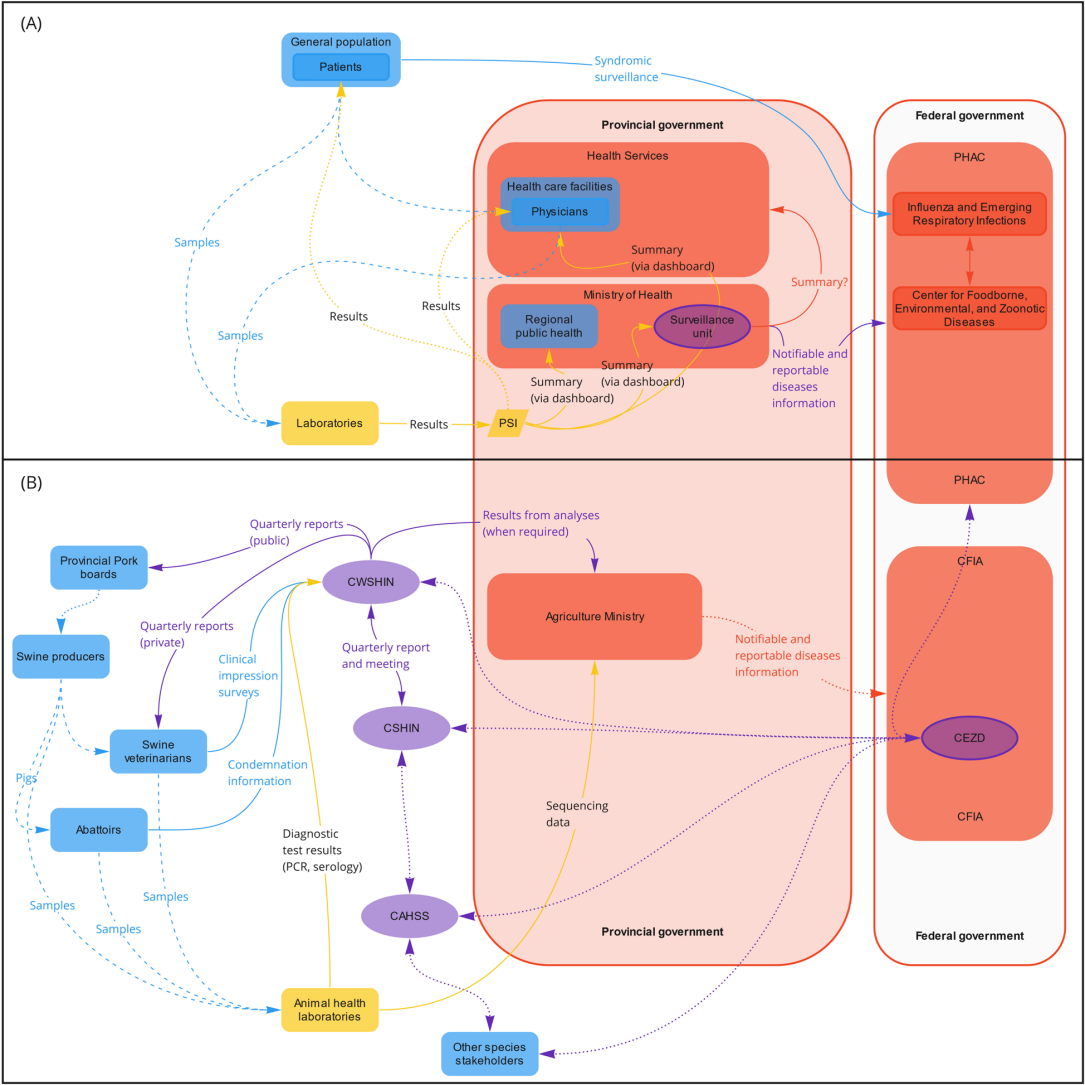
Structural communication links identified for human (**A**) and swine (**B**) influenza surveillance in Alberta. Information is usually shared from left to right: from laboratories, through the Provincial Surveillance Initiative system, back to the patients and referring physicians, as well as surveillance groups within the provincial government. Some information (anonymized) also flows to the federal government: from veterinarians, laboratories, and abattoirs to Swine Health Intelligence Networks (e.g., CWSHIN and CSHIN) to governments (provincial and federal). There is also publicly available information shared by the Community for Emerging and Zoonotic Diseases to various stakeholders (from right to left). Dashed lines: samples; Full lines: data; Dotted lines: results/summaries. Blue: field stakeholders; Yellow: laboratories; Purple: intelligence; Red: government. *CAHSS* Canadian Animal Health Surveillance System; *CEZD* Community for Emerging and Zoonotic Diseases; *CFIA* Canadian Food Inspection Agency; *CSHIN* Canadian Swine Health Intelligence Network; *CWSHIN* Canada West Swine Health Intelligence Network; *GPHIN* Global Public Health Intelligence Network; *PHAC* Public Health Agency of Canada; *PSI* Provincial Surveillance Initiative

Figure 1B shows communication channels as they flow (from left to right) in Alberta. Influenza virus in pigs is provincially notifiable^2^ in Alberta, British Columbia and Saskatchewan, but it is not federally notifiable. At the regional level, the Canada West Swine Health Intelligence Network (CWSHIN) combines and analyzes the data from British Columbia, Alberta, Saskatchewan, and Manitoba. It includes clinical impression surveys from swine veterinarians, laboratory diagnostic data from provincial and university laboratories (presence of pathogens, or serological or anatomical indicators), and condemnation rates from federally inspected slaughterhouses [11]. Once analyzed, the information is shared quarterly with veterinarians (reports, as private communications) and producers (reports, as public communications) and, when requested to address animal, human, or ecosystem concerns, with provincial governments (Fig. 1B). While some analyzed data are publicly available via reports for producers, our participants stated that there are no other direct communication channels between the CWSHIN and public health stakeholders. However, the regional surveillance networks (CWSHIN, Ontario Animal Health Network, and Réseau d’alerte et d’information zoosanitaire) are part of the Canadian Swine Health Intelligence Network (CSHIN) and the CAHSS, which include members from the National and Provincial pork councils, veterinary colleges, diagnostic laboratories, provincial governments, CFIA, Agriculture and Agri-Food Canada (AAFC), Public Health Agency of Canada (PHAC), and national and regional veterinary organizations and networks.

#### Routine communication channels for the surveillance of influenza virus infection in the human sector in Alberta

In Alberta, laboratory data flow through a single laboratory information system (Provincial Surveillance Initiative; PSI), and information is automatically transmitted to stakeholders (e.g., physicians, patients, and surveillance units within the Ministry of Health; Fig. 1A) via an online platform. This system allows the linkage of clinical and epidemiological data with laboratory data at the provincial level.

The data about influenza collected by the healthcare system are gathered provincially and then anonymized and shared with FluWatch, a national surveillance program for influenza and influenza-like illnesses (ILI) [18]. The program monitors, inter alia, health care admission for influenza or ILI, laboratory-confirmed detection, syndromic surveillance, outbreak and severe outcome surveillance, and vaccine coverage; it shares weekly reports online [18]. At the provincial and federal levels, we were unable to identify other communication channels for providing human influenza surveillance information to animal health stakeholders.

#### Routine communication channels between the swine and human sectors for the surveillance of influenza in Alberta

Many swine health surveillance stakeholders are members of the Community for Emerging and Zoonotic Diseases (CEZD). This multidisciplinary network of public and animal health experts from government, industry and academia was developed to support early warning, preparedness, and response for animal emerging and zoonotic diseases [20]. Open source signals are extracted automatically via the Knowledge Integration using Web Based Intelligence (KIWI) [21] and manually by the CEZD core team (CFIA employees). This team assesses signals daily, with rolling support from volunteer members and from expert partners from federal and provincial governments, academia, and industry when needed. Signals are then shared with the CEZD community, including through immediate notifications of important disease events, group notifications and pings, quarterly sector-specific intelligence reports and weekly intelligence reports.

Although CEZD was growing during the 2020-2021 period [22], membership is voluntary, as it is the case for CAHSS. Moreover, both networks cover multiple species and diseases, which serves to maximize the reach of the communities but can result in an overwhelming amount of information for members whose main interest is in another sector, such as human or ecosystem health. This large amount of information primarily relevant to other sectors can lead members to leave or not join these two networks.

### Communication channels between sectors during a human case of swine influenza in Alberta

In all cases where a new influenza subtype, including an animal influenza subtype, is identified from a human case, this must be reported to the World Health Organization (WHO) under the IHR [23]. In Canada, PHAC is the body responsible for notifying the WHO of such cases. We examined the IHR-reportable case of a human infected with an animal influenza subtype identified in October 2020 in Alberta. The event we examined happened during an exceptional period for ILI as it was less than a year after the WHO declared the 2019 novel coronavirus disease (COVID-19) a global pandemic. At that time, influenza activity remained below average, most ILI symptoms were due to COVID-19 cases, and most public health and human health resources were dedicated to managing the pandemic [24].

**Fig. 2:**
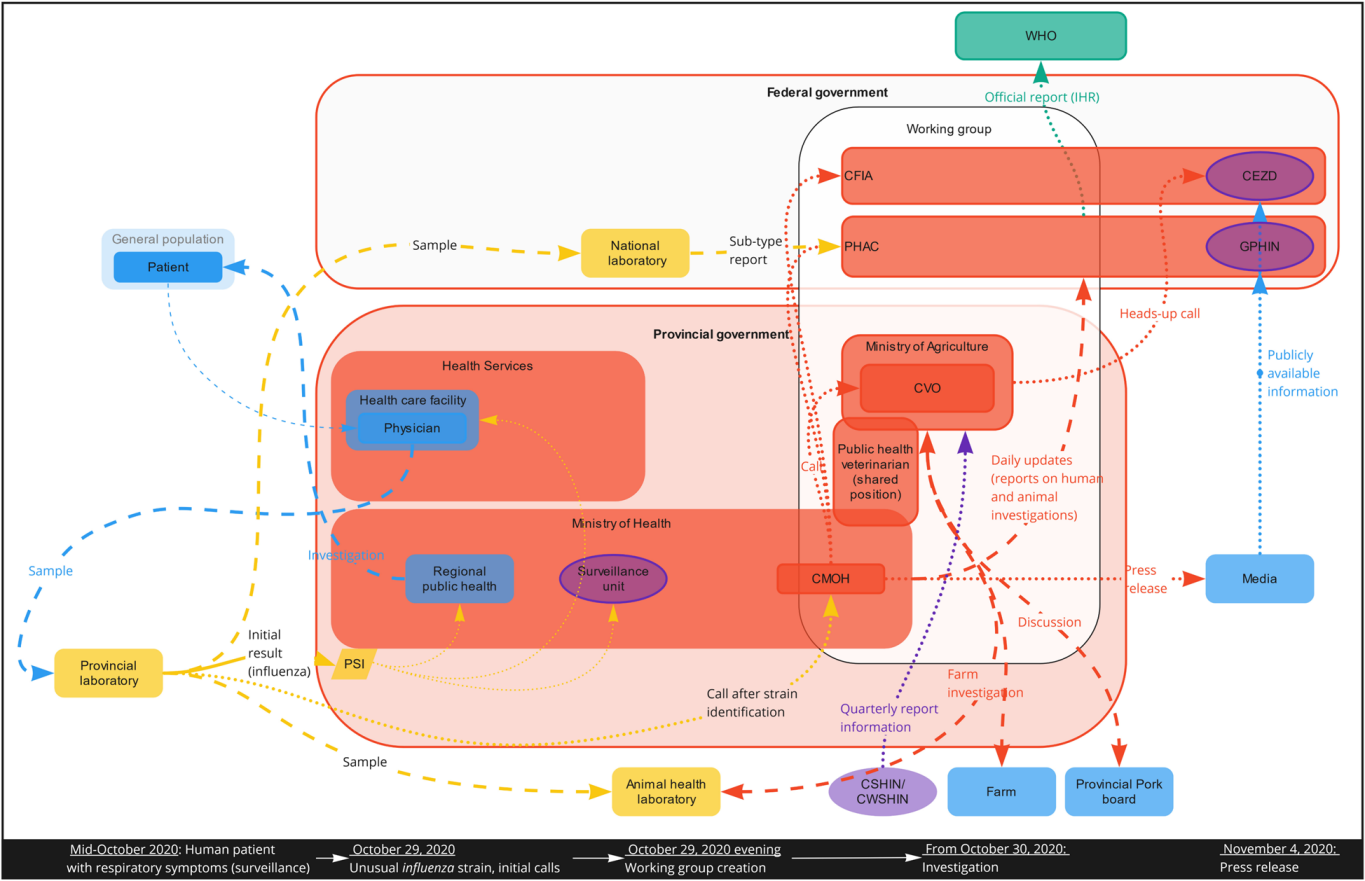
Timeline and communication links during the human influenza A H1N2v case in Alberta. Dashed lines: samples; Full lines: data; Dotted lines: results/summaries Blue: field stakeholders; Yellow: laboratories; Green: intelligence; Red: government, Purple: international. *CEZD* Community for Emerging and Zoonotic Diseases; *CFIA* Canadian Food Inspection Agency; *CMOH* Chief Medical Officer of Health; *CSHIN* Canadian Swine Health Intelligence Network; *CVO* Chief Veterinary Officer; *CWSHIN* Canada West Swine Health Intelligence Network; *GPHIN* Global Public Health Intelligence Network; *PHAC* Public Health Agency of Canada; *PSI* Provincial Surveillance Initiative; *WHO* World Health Organization

In the case we investigated, the influenza subtype identified through sequencing performed at a provincial laboratory on October 29, 2020 (Fig. 2) in the human case was a variant similar to a swine influenza virus (A H1N2v). Samples from the human case were then sent to a reference laboratory, the National Laboratory of Microbiology (NLM) of PHAC, for confirmation. A provincial laboratory stakeholder also contacted a University Animal Health Laboratory colleague and sent the human sample in parallel for sequencing and confirmation that the variant was a swine virus.

Because the human case was IHR reportable and had potential for high visibility, the provincial laboratory immediately contacted the Alberta Chief Medical Officer of Health (CMOH). Provincial and federal government stakeholders (Alberta Health, Alberta Health Services, Alberta Agriculture and Forestry, PHAC, CFIA) were called to an evening meeting to raise awareness and ensure that the situation was managed in a way that satisfied provincial, federal and international obligations. This “H1N2v working group” was put in place quickly, apparently following the initiative of the Alberta CMOH (not confirmed as no interview was conducted with the initiators of this working group).

The information PHAC received through formal communication channels (e.g., from the NLM) took longer compared to the original call by the CMOH and the H1N2v working group. For this study, we did not have access to the guidelines in place for such an event, and it is unclear if the other stakeholders (provincial Ministry of Agriculture and CFIA) were officially needed to be involved.

Because swine influenza is endemic in the porcine population and this case was of importance for human health, the provincial public health stakeholders led the initiative, with the support of other stakeholders. The H1N2v working group met at least twice following the initial meeting. Additionally, follow-up data was gathered at the provincial level via multiple channels (public health, animal health, epidemiological, and laboratory investigations), and findings from the various investigations were shared with PHAC daily for a week and then weekly for two additional weeks. Information sharing between provincial and federal public health entities seemed to follow a formal process, but while we had access to the communication template, none of the interviewed participants had information about the structure supporting this initiative.

In the meantime, regional public health partners (within Alberta Health Services) were mandated to conduct the field investigation for the human case and its contacts with humans and pigs, supported by Alberta Agriculture and Forestry and stakeholders from the swine sector (e.g., Alberta Pork). The investigation’s goal was to clarify whether the infection was contracted from animal-to-person (directly or indirectly) or person-to-person. The public health investigation, the available information about swine influenza in the province (obtained from the CSHIN report), and the farm investigation performed in collaboration with an Animal Health Laboratory all provided supporting data.

The human and animal investigation data were collected by multiple stakeholders. The communication of results followed formal structures through Alberta Health (case, laboratory and epidemiological investigation results) and Alberta Agriculture and Forestry (farm investigation results) and were ultimately shared with PHAC. Interviewees reported that coordination of the two provincial ministries in this case was facilitated by the public health veterinarian, whose position is shared between the two ministries. Interviewees also said that in the investigation’s early stages, the swine sector’s participation in the farm investigation (an informal channel, via Alberta Pork) facilitated communication between the government and the farm involved. This highlights the importance of strong formal and informal government-industry relationships, which ensured that farmers and stakeholders trusted the system enough to support the investigation.

While the investigation was still ongoing and a clearer picture of the case and its transmission was emerging, a decision was made to make the information public. Our interviews did not identify the process leading to this decision, but six days after the initial notification to the government officials, an Alberta CMOH press release was distributed, with information stating there was limited risk for the general population. This now-public information was then identified by at least two Canadian event-based surveillance (EBS) systems that distributed the information to their communities. One of the EBS interviewees mentioned, however, that they received an email from the Alberta Agriculture and Forestry the night before the press release so they could prepare for it and have a notification ready to be shared. This informal communication channel seemed to arise from a preexisting relationship between stakeholders involved.

### Encouraging and inhibiting elements involved in OH communication

The communication channels evident in our case study allowed us to identify elements involved in the information flow between animal and human health stakeholders (Table 3). Identifying what information needed to be shared between sectors was influenced by actors’ understanding of the evidence needed to trigger decisions and actions. During the surveillance phase, information was available online from the animal health (CWSHIN, CSHIN, CAHSS, CEZD) and human health (FluWatch, Global Public Health Intelligence Network) sectors. However, it was difficult to quantify how much these sources were used by different stakeholders. We identified little other communication between animal and human health stakeholders during this phase. Stakeholders reported having very limited time and resources to consult and use information from other sectors, suggesting a need for policies and structural integration of OH. For example, having a public health veterinarian appointed at both the provincial agriculture and health ministries was mentioned as a key element facilitating communication and coordination (Quote 1).

**Table 3:** Mechanisms identified within a case study of influenza surveillance systems and of a zoonotic outbreak

	**Stakeholders mentioned information was shared**		**Stakeholders mentioned information was not shared**	
**Elements**	**In a situation where the element was present**	**In a situation where the element was absent**	**In a situation where the element was present**	**In a situation where the element was absent**
Identifying information to share				
Understanding of other sectors’ needs	Surveillance: - Actions are taken to increase human and ecosystem health stakeholders in CEZD membership. Outbreak: - Animal health stakeholders shared surveillance information and supported field investigation.	Surveillance: - Reports from animal health (CWSHIN, CSHIN, CAHSS, CEZD) and human health (FluWatch, GPHIN) is available online.		Surveillance: - Information is not systematically shared between sectors.
Type: information (vs data)	Surveillance: - Analyzed data (information) is shared or available in reports.			Surveillance and outbreak: - Privacy and ethical barriers to share data within (e.g., provincial to federal) and between sectors.
Sharing information				
Presence of communication channels	Outbreak: - The shared position between the Alberta Ministries of Agriculture and Health was a facilitator for coordinating.			Surveillance: - Complicated information sharing systems is a barrier to communication between sectors.
Type: formal channel (vs informal)		Outbreak (unclear): - Guidelines for an internationally reportable human disease needed the involvement of PHAC, but it is uncertain if the participation of animal health stakeholders was prescribed too.	Surveillance and outbreak: - Direct communication channels (phone call) were faster than formal channels.	Surveillance: - Lack of formal interaction within and between some human and animal health systems can result in missed information transfer.
Trust	Surveillance: - Information sharing within and between human, animal, and ecosystem health stakeholders requires trust.			
Identifying information to share and sharing information				
Resources				Surveillance: - Restricted budgets for some programs, impeding ability to connect with other organizations/programs, and impacting resilience of infrastructure and health systems. - There is a need for additional medical expertise resource and policy to be able to integrate One Health to policies.

*CEZD* Community for Emerging and Zoonotic Diseases, *CWSHIN* Canada West Swine Health Intelligence Network, *CSHIN* Canadian Swine Health Intelligence Network, *CAHSS* Canadian Animal Health Surveillance System, *GPHIN* Global Public Health Intelligence Network, *PHAC* Public Health Agency of Canada

Quote 1.

“When the pandemic started, we had our public health veterinarian position empty. […] That position is essentially fully dedicated to working between the two ministries [Agriculture and Health]. It [the impact of this vacancy] showed itself in terms of just some gaps for them working on things without consulting us, but then [when] that position was filled and the other relationships were in place, everything just went really smoothly. […] it demonstrated the importance of those relationships and… having a good liaison between the two departments.”

During the outbreak, surveillance, laboratory, and industry information on swine influenza was quickly available to human health stakeholders. Animal health stakeholders, however, noted that the communication was, unfortunately and as in many cases, only one way. Barriers to within- and cross-sector communication included complicated or lacking communication channels. In our case study, there was a formal channel between the provincial and federal government due to the IHR requirements, but this is not the case for non-IHR-reportable zoonotic diseases. Moreover, the CMOH’s phone call to other stakeholders to create the H1N2v working group occurred faster than the formal communication channels.

Established professional connections facilitated information flow between stakeholders who understood each other’s needs and interests. While a lack of formal channels was identified as a pitfall due to potentially missed communication opportunities, many participants mentioned that established, informal relationships and networks facilitated information sharing – both the assessment of how much and what type of information to share and with whom it should be shared. Informal and formal communication channels were also affected by privacy and ethical concerns. Raw data, usually confidential, obtained from either the animal or human health sectors cannot easily be shared, adding to the complexity of formal communication channels. Analyzed or summarized data (i.e., information) were easier for both animal and human health sectors to share in reports or online platforms.

Trust, which can be defined as the perceived benevolence, integrity, competence and predictability of the other [25], was identified as the foundation for good communication among different stakeholders, whether via formal or informal channels. Here, previous interactions between stakeholders likely served as a basis for trusting that the person receiving the information would be kind, competent, honest, and predictable when using it. From the perspective of animal health stakeholders, however, trust was more difficult: the perceived anthropocentric perspective of health initiatives, including OH initiatives [26], created fear that shared information might not be reciprocated and would have negative repercussions on animals and producers (Quote 2).

Quote 2.

“You need to build trust and it takes a long time […] you need to build that trust with individual livestock sectors, that human health is not going to destroy the sector^a^. The [animal health] sector is generally very cautious because their perspective is very rarely considered […] if you have a human pathogen […] in livestock and it can potentially transfer to people, all the burden is very often on the livestock. […] Human health has a lot of resources and animal health doesn't, but they get all [the burden]. It's a matter of who [has] the cost and who's benefiting.”

^a^While the stakeholder interviewed did not give additional details, they could have been referring to the case of a herd where an emerging influenza virus (H1N1v) was identified, which resulted in depopulation of the herd [4]. This was a severe consequence for the farmer, while the source of the virus was determined to be an infected human. They could also have been referring to the possibility of zoonotic events decreasing the marketability of meat because of public perception or export restrictions. This was unfortunately not discussed further in the interview

**Fig. 3:**
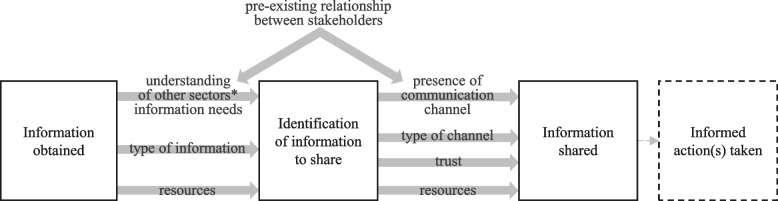
Elements linking the steps involved between obtaining information and sharing information to another health sector ^*^The two sectors examined in the present case study are animal health and human health

Interviewees suggested that information sharing requires two main steps: (1) identifying what information must be shared and (2) sharing that information with another sector (Fig. 3). Once stakeholders within a sector had information, the first step was identifying what should and can be shared, with whom, and through what channels. This could be facilitated or impeded by actors’ perceptions of other sectors’ needs, the type of information that is available, and the resources available. For sharing information itself, both the presence and type of communication channels were critical for external information sharing with other sectors – but so were trust and the availability of resources. Preexisting relationships among stakeholders also shaped actors’ understanding of each other’s needs, the presence of informal channels, and trust.

## Discussion

This case study highlights the complex communication structures for influenza surveillance and response in both human and animal health sectors and the limited links between these sectors. It illustrates the importance of rapid and open communication channels between these sectors in both surveillance and response contexts. While day-to-day surveillance aims to detect and monitor influenza viruses, the detection of a human case harboring an animal subtype resulted in a specific response, which triggered different channels. While information flows through formal and informal channels, trust is a critical component in all types of communication: between animal and human health actors, between government and livestock sectors, and between international, federal, provincial and territorial, and regional jurisdictional levels. Developing and maintaining relationships among stakeholders requires time and resources but is essential for mutual understanding of information needs and rapid communication.

While previous studies found that communication is a key factor for OH initiatives [27, 28], we were able to identify processes that were in place when good communication occurred. These findings offer a new perspective that could be useful to many surveillance and response programs. For example, networks and structures are often described for influenza programs, but the communication channels and information flow are not detailed [29–31]. This is a gap that would be useful to address, especially as we found that while formal structures are necessary, informal structures allow for quicker and more efficient communication and coordination.

### Limitations

While the findings from this study highlight key elements of good One Health communication, the retrospective interpretive process tracing of a case study has certain limitations. First, our study was based on an influenza case, for which there are established surveillance systems and protocols [11, 18, 23]. This likely contributed to the effective response but also influenced our findings. We think this could have hidden or minimized some of the challenges faced by stakeholders regarding OH communication. For example, in the case of a disease that has no formal surveillance system reporting guidelines, challenges might be different. Second, we purposively selected a “success story” to illustrate what happens when OH communication goes well. Due to this retrospective selection of our case study, we suspected that communication and coordination went well prior to starting the project. This could have influenced our findings, and it is possible that we would have had different conclusions if we used a case study for which communication and coordination were suboptimal. To mitigate this, we designed the study with an interpretive approach focusing on the interviewees’ own perspectives, with as little preconceived bias as possible [8]. Third, the case we chose happened during the COVID-19 pandemic. The high focus on ILI during this period could have strengthened some communication channels. For example, many resources were deployed to manage the pandemic, which may have facilitated communication and integration among sectors. Fourth, this could have also affected the stakeholders who agreed to participate in the interviews, which were conducted at a later stage of the pandemic. Indeed, six human health stakeholders who had key positions in this case declined or did not reply to our invitation, and our findings lack their perspective. It is possible that more communication channels between human and animal health exist, but we were not able to identify them. The barriers and limitations we identified are possibly different for stakeholders in the human health sector; additional research related to the involvement of these actors in OH communication would be beneficial. Fifth, due to the limited resources available for this project, the focus of the case study (swine and public health), and the process we used to identify the stakeholders to interview, we did not identify stakeholders from the environment and wildlife health sector, or from other livestock health sectors (e.g., poultry). This is, in itself, a finding, highlighting the limited communication channels among these stakeholders. It is however unclear if our findings about facilitators and barriers are generalizable to all sectors.

## Conclusion

While additional research, including larger comparative studies, is needed, our findings highlight the importance of investing time and resources in supporting relationship building, as well as formal communication mechanisms, among stakeholders in the human, animal, and ecosystem health sectors.

## Data Availability

No datasets were generated or analysed during the current study.

## Abbreviations

AAFC: Agriculture and Agri-Food Canada
CAHSS: Canadian Animal Health Surveillance System
CFIA: Canadian Food Inspection Agency
CMOH: Chief Medical Officer of Health
CSHIN: Canadian Swine Health Intelligence Network
CWSHIN: Canada West Swine Health Intelligence Network
CEZD: Community for Emerging and Zoonotic Diseases
EBS: Event-based surveillance
IHR: International Health Regulations
ILI: Influenza-like illnesses
KIWI: Knowledge Integration using Web Based Intelligence
NLM: National Laboratory of Microbiology
OH: One Health
PHAC: Public Health Agency of Canada
PSI: Provincial Surveillance Initiative
WHO: World Health Organization

## Footnotes

1 While the One Health approach should in principle engage stakeholders from human, environmental, and animal health sectors, the scope of the current study focused on public health and livestock health sectors. Throughout the manuscript, we used public and human health interchangeably, as for animal and livestock health.

2 Reportable and notifiable diseases must be reported to federal and/or provincial governments. Reportable diseases generally pose significant threats to animal health, public health, or food safety, while notifiable diseases are monitored for trends or changes.
